# Paracentesis complication rates and use of ultrasound: impact of a point-of-care ultrasound training course in the veterans affairs healthcare system

**DOI:** 10.1186/s12909-025-07656-z

**Published:** 2025-08-12

**Authors:** Robert Nathanson, Rahul Khosla, Rebecca Theophanous, Zahir Basrai, Abdulkareem A. Agunbiade, Christopher Schott, Jeremy Boyd, Michael J. Mader, Kevin J. Murray, Jacqueline A. Pugh, Erin P. Finley, Nilam J. Soni

**Affiliations:** 1 South Texas Veterans Health Care System, Medicine Service, San Antonio, Texas United States; 2 Division of Hospital Medicine, Department of Medicine, University of Texas Health San Antonio, 7703 Floyd Curl Dr, MC 7982, San Antonio, 78229 Texas United States; 3 Department of Pulmonary, Critical Care and Sleep Medicine, The George Washington University, Washington, DC United States; 4 Pulmonary and Critical Care Medicine, Veterans Affairs Medical Center, Washington, DC United States; 5 Emergency Medicine, Durham VA Healthcare System, Durham, North Carolina United States; 6 Duke University School of Medicine, Department of Emergency Medicine, Durham, North Carolina United States; 7 Emergency Medicine, VA Greater Los Angeles Healthcare System, Los Angeles, California United States; 8 Department of Emergency Medicine, David Geffen School of Medicine at UCLA, Los Angeles, California United States; 9 VA Pittsburgh Health Care Systems, Critical Care Service, Pittsburgh, Pennsylvania United States; 10 University of Pittsburgh, Departments of Critical Care Medicine and Emergency Medicine, Pittsburgh, Pennsylvania United States; 11 Vanderbilt University Medical Center, Department of Emergency Medicine, Nashville, Tennessee United States; 12 VA Tennessee Valley Healthcare System—Nashville, Department of Emergency Medicine, Nashville, Tennessee United States; 13 South Texas Veterans Health Care System, Research Service, San Antonio, Texas United States; 14 Louis Stokes Cleveland VA Medical Center, Medicine Service, Cleveland, Ohio United States; 15 Case Western Reserve University School of Medicine, Department of Medicine, Cleveland, Ohio United States; 16 VA Greater Los Angeles Healthcare System, Center for the Study of Healthcare Innovation, Implementation, and Policy (CSHIIP), Los Angeles, California United States; 17 University of Texas Health San Antonio, Division of Hospital Medicine, San Antonio, Texas United States; 18 University of Texas Health San Antonio, Center for Research to Advance Community Health (ReACH), San Antonio, Texas United States

**Keywords:** Point of care, Ultrasound, Education, Procedures, Clinical outcomes

## Abstract

**Background:**

Point-of-care ultrasound (POCUS) training courses have been shown to increase knowledge and skills among physicians, but few studies have examined their impact on clinical outcomes. We assessed the frequency of ultrasound usage and complication rates of paracentesis after implementing a POCUS training course in the Veterans Affairs (VA) health care system.

**Methods:**

A retrospective observational study was conducted of VA medical centers that participated in a POCUS training course (“trained facilities”) versus matched control facilities. Rates of paracentesis performed in non-radiology settings with and without ultrasound guidance and procedural complications were collected from the VA corporate data warehouse (CPT and ICD-10 procedure and diagnosis codes) and pre- and post-course surveys of course participants. A best fit linear regression line was established for quarterly rates of each group and the y-intercept and slope of each line was compared.

**Results:**

Data were compared from 16 trained versus 32 matched control facilities where 10,375 and 22,103 paracenteses were performed, respectively, from October 2015 to March 2025. At baseline, ultrasound guidance was used less frequently in trained versus matched control facilities (39% vs. 78.3%, *p* < 0.0001). However, trained facilities demonstrated a greater quarterly increase in ultrasound use (1.20% vs. -0.14% per quarter, *p* < 0.0001). By the end of the study, trained facilities surpassed control facilities in use of ultrasound guidance (84.6% vs. 73.0%, *p* < 0.001). The overall complication rate was low (2.4 per 1,000 procedures) and there was no significant difference in trends between trained and matched control facilities.

**Conclusions:**

Participation in a national POCUS training course was associated with increased use of ultrasound guidance for paracentesis but not with statistically significant changes in complication rates. Further study is warranted to explore effect of POCUS training on procedural outcomes of paracentesis.

**Supplementary Information:**

The online version contains supplementary material available at 10.1186/s12909-025-07656-z.

## Background

It is estimated that over 150,000 paracenteses are performed annually in the United States [1]. Though important for patient care, these procedures carry significant risks of complications that increase morbidity and mortality [2–4]. Point-of-care ultrasound (POCUS) use to guide paracentesis has become standard of care to avoid unnecessary procedure attempts, improve procedure success rates, and reduce complication rates, costs, and length of stay [4–7]. Most specialties now incorporate POCUS into clinical practice, and a national survey of Veterans Affairs (VA) medical centers reported a marked rise in its use across specialties from 2015 to 2020 [8–10]. 

Despite its growth, limited training remains a significant barrier to POCUS use. While 80–89% of specialty chiefs in the VA express interest in POCUS training, only 34–37% of specialty chiefs report having a process to train their physicians [8–10]. To help address this barrier, the VA healthcare system launched a national POCUS training course in 2016. The course significantly improved participants’ POCUS knowledge and skills, with retention observed at 8 months post-course [11]. However, few studies have evaluated the effect of standardized POCUS training on patient outcomes.

We hypothesized that facilities participating in the VA POCUS training course would demonstrate increased use of ultrasound guidance for paracentesis and reduced complication rates compared to matched controls.

## Methods

### Study design and setting

We conducted a retrospective observational study to evaluate the change in ultrasound usage and complication rates for paracentesis after implementing a national POCUS training course in the VA healthcare system. The University of Texas Health San Antonio Institutional Review Board deemed this project to be nonregulated research exempt from review (Protocol Number: HSC20160445N).

### Participants and study sites

We invited 21 VA medical centers that met inclusion criteria to participate in the POCUS training course. Inclusion criteria were: (1) Chief of Staff and Service Chief approval to allow clinicians at their facility to participate, (2) financial support from the Chief of Staff to fund course participants’ travel to the VA’s National Simulation Center in Orlando, Florida, and (3) availability of ≥ 2 portable ultrasound machines in the work areas of the target specialties at the facility [11]. Sixteen facilities that met inclusion criteria and sent ≥ 4 clinicians to the training were classified as “trained facilities.” The matched control facilities (*n* = 32) were similar in size, complexity, and reported POCUS use who had ≤ 1 clinician participate in the POCUS training course. To ensure all study facilities were starting with similar baseline POCUS experience, Chiefs of Staff of the 16 trained facilities and 32 matched control facilities reported that POCUS was in use in ≥ 4 different specialties other than interventional radiology at their facility before October 2016.

### Intervention

The structure, curriculum, and effectiveness of the VA POCUS training course have been previously published [11]. Courses were held from October 2016 to March 2020 at the VA’s National Simulation Center in Orlando, Florida, and targeted physicians specializing in emergency, hospital, and critical care medicine with minimal prior ultrasound training. The course agenda was similar to other standardized POCUS CME courses and covered multi-system diagnostic POCUS applications and ultrasound-guided procedures. During the 2.5-day course, participants received a 45-minute lecture on ultrasound-guided procedures including paracentesis. Participants subsequently received hands-on skills training for identification of abdominal free fluid, paracentesis site marking, and pre-procedural evaluation of blood vessels on the abdominal wall with color flow Doppler, using both live and simulation models.

### Data sources and variables

The VA corporate data warehouse was searched for CPT and ICD-10 procedure codes for paracentesis from trained and matched control facilities from October 2015 through March 2025 (Additional File 1) which included 1 year of baseline data prior to course launch. Ultrasound guidance was assessed using CPT codes, as ICD-10 procedure codes do not specify use of imaging guidance. Complications (bleeding and bowel injury) occurring within five days of paracentesis in acute care/hospital, emergency, or outpatient settings were identified via ICD-10 diagnosis codes. We excluded procedures performed by radiology but separately tracked ultrasound guidance rates in radiology settings as a validation step. Course participants also completed pre- and 8-month post-course surveys (Additional File 2) which included questions about POCUS use, comfort level (0-100 visual analog scale), and complications of paracentesis [11]. 

### Outcomes

Primary outcomes were the proportion of paracenteses performed with ultrasound guidance and complication rates per 1,000 procedures. Secondary outcomes included changes in course participants’ self-reported ultrasound usage and comfort with ultrasound-guided paracentesis, as well as paracentesis complications.

### Statistical analysis

We calculated quarterly proportions of ultrasound-guided paracenteses and complication rates for trained and matched control facilities. Best fit linear regression lines were generated to assess trends over time. Differences in the y-intercept and slope of each line were compared using a generalized linear model (two-factor ANOVA with interaction term). We used a significance threshold of 0.05 for ultrasound guidance and 0.10 for complication rate comparisons due to the low number of complications from paracentesis. For secondary outcomes, the McNemar test was used to compare changes in use of ultrasound for paracentesis and paracentesis-related complications within the past year, as reported by participants. Changes in comfort level were analyzed using a two-sided paired-samples t-test. A significance threshold of 0.05 was used for all secondary outcome analyses.

## Results

Characteristics of trained and matched control facilities are summarized in Table 1 and provider survey data are summarized in Table 2. There were no significant differences in baseline characteristics between the trained and matched control facilities, including general POCUS use, use of ultrasound guidance for paracentesis in the target specialties, and availability of institutional infrastructure (local POCUS training, policies, privileging criteria, and image archiving) (Table 1). A total of 148 physicians from the 16 trained facilities completed the course between October 2016 and August 2019, for an average of 9 course participants per facility. The 32 matched control facilities had a total of 8 course participants attend the POCUS training course between October 2017 and March 2020, with 75% of matched control facilities sending no clinicians to participate in the course. The majority of participants came from hospital medicine (39%), emergency medicine (18%), and critical care medicine (15%), with fewer participants representing anesthesiology, pulmonary medicine, and other specialties [11]. During the study period, 10,375 and 22,103 paracenteses were performed in non-radiology settings at trained and matched control facilities, respectively (Additional File 3).

**Table 1:** Characteristics of trained and matched control facilities

Characteristic	Trained Facilities (*N* = 16)	Matched Facilities (*N* = 32)	*p*-value
**Size**			
<100 beds (Medicine + Surgical) >100 beds (Medicine + Surgical)	7 (44%) 9 (56%)	16 (50%) 16 (50%)	NS
**Region**			
Northeast Midwest South West	2 (13%) 3 (19%) 7 (44%) 4 (25%)	4 (13%) 8 (25%) 14 (44%) 6 (19%)	NS
**Location**			
Urban	16 (100%)	32 (100%)	NS
**VHA Facility Complexity Level** ^**1**^			
High Low	16 (100%) 0 (0%)	32 (100%) 0 (0%)	NS
**POCUS Use**			
POCUS used in hospital medicine	13 (81%)	20 (63%)	NS
POCUS used in critical care medicine	15 (94%)	26 (81%)	NS
POCUS used in emergency medicine	16 (100%)	28 (88%)	NS
POCUS used for paracentesis^2^	70%	57%	NS
**Training and Infrastructure**			
Process to obtain POCUS training^3^	3 (19%)	4 (13%)	NS
≥1 Facility-wide policy for POCUS^4^	7 (44%)	18 (56%)	NS
Privileging criteria	6 (38%)	9 (28%)	NS
Image archiving	7 (44%)	6 (19%)	NS
Quality assurance	6 (38%)	6 (19%)	NS

^1^High complexity facilities had high patient volumes, patient risk, specialists, teaching, and research. Low complexity facilities had medium to low patient volumes, patient risk, and some to little teaching or research

^2^Proportion of hospital medicine, critical care medicine, and emergency medicine sections that reported use of POCUS for paracentesis by their clinicians

^3^Service chiefs of hospital, critical care, and/or emergency medicine reported availability of a local process to obtain POCUS training

^4^Chiefs of staff reported availability of formal policies on POCUS credentialing/privileging and/or required demonstration of competency for POCUS use

Abbreviations: POCUS, point-of-care ultrasound; VA, Veterans Affairs; NS, not significant

**Table 2:** Pre- and Post-course frequency of ultrasound usage and complications of paracentesis per course participants

Survey Question	Pre-Course	Post-Course (8 Months)	*p*-value
POCUS used for paracentesis	39/115 (34%)	52/115 (45%)	0.012
Comfort level with US-guided paracentesis^1^	30.0	51.4	< 0.001
Paracentesis complications within past 1 year	5/115 (4.3%)	1/115 (0.9%)	0.046

^1^Clinicians were asked to rate their comfort with US-guided paracentesis on a scale of 0 (not comfortable at all) to 100 (very comfortable)

Abbreviations: POCUS, point-of-care ultrasound; VA, Veterans Affairs; US, ultrasound

### Ultrasound guidance

**Fig. 1:**
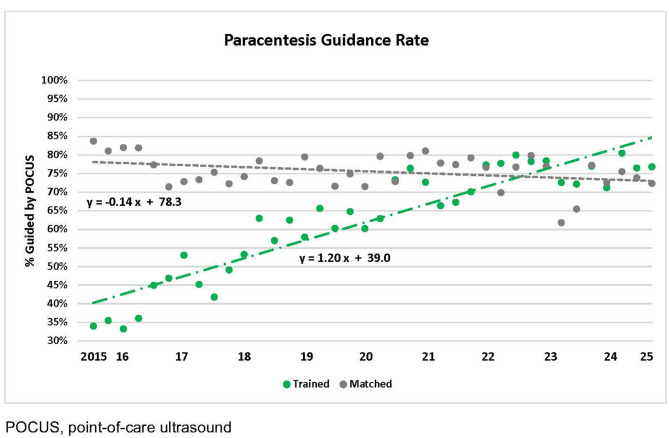
Ultrasound Guidance Rates for Paracentesis from October 2015 to March 2025

Trained facilities had lower baseline use of ultrasound guidance compared to matched control facilities (39.0% vs. 78.3%, *p* < 0.0001), but had greater improvement over time (1.20% per quarter vs. -0.14% per quarter, *p* < 0.0001) (Fig. 1). Radiology consistently performed paracentesis with ultrasound guidance from 2015 to 2025 (Additional File 4). By March 2025, trained facilities surpassed matched control facilities in the use of ultrasound guidance (84.6% vs. 73.0%, *p* < 0.0001). Furthermore, participant surveys confirmed increased confidence (30 vs. 51 on a 100-point scale; *p* < 0.001) and ultrasound usage (34% vs. 45%; *p* = 0.012) for paracentesis 8 months post-course (Table 2).

### Complication rates

**Fig. 2:**
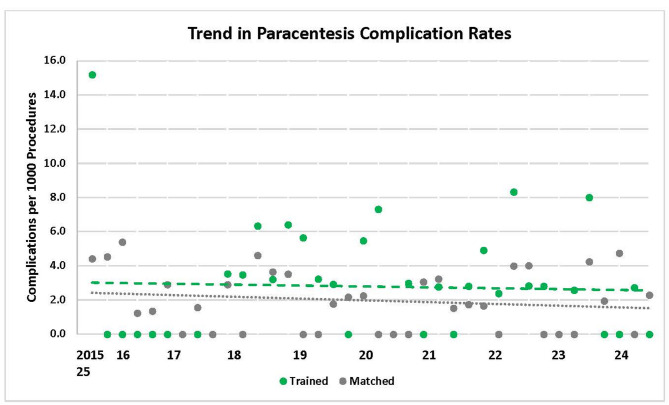
Paracentesis Complication Rates from October 2015 to March 2025

Overall complication rates were low (2.4 per 1,000 procedures). All recorded complications were bleeding-related and no bowel injuries were identified (Additional File 5). Both trained and matched control facilities showed similar flat trends in complication rates with no significant differences between the groups over time (Fig. 2). Surveyed course participants reported a decline (4.3% vs. 0.9%; *p* = 0.046) in complications post-course (Table 2).

## Discussion

To the best of our knowledge, this is the first study to assess the impact of a national POCUS training course on use of ultrasound guidance and procedural complication rates for paracentesis. Facilities supporting physician participation in a standardized POCUS training course was associated with an increase in use of ultrasound guidance for paracentesis, and trained facilities had a significantly higher rate of ultrasound guidance by the end of the study period. Further, course participants reported a significant increase in comfort and use of ultrasound guidance for paracentesis at 8 months post-course. However, paracentesis complication rates were low, and POCUS training did not significantly affect complication rates.

Our study highlights the importance of POCUS training to promote adoption of ultrasound guidance for bedside procedures. Prior single-center studies have demonstrated that simulation-based education for paracentesis leads to improved procedural skills on task-trainers and increased knowledge [12–14]. However, using the Kirkpatrick framework for educational interventions (Level 1-Reactions, 2-Learning, 3-Behavior, 4-Results), it is difficult to demonstrate level 3 and 4 outcomes [i.e., learners are using skills and knowledge they were taught (Level 3, Behavior) and learning resulted in improved patient outcomes (Level 4, Results)] [15]. While our retrospective study is unable to prove direct causation, the results demonstrate an association between participation in a national POCUS training course with increased use of ultrasound guidance for paracentesis (Level 3). Decreased procedural complication rates (Level 4) was not observed in part due to low complication rates of bleeding and reduced statistical power.

How a national POCUS training course impacts facility-level outcomes remains a subject of debate. While course participants demonstrated increased POCUS use for paracentesis, it is possible they pursued additional training which may have further influenced outcomes. Prior studies suggest that clinicians who use POCUS are more likely to seek further training, potentially due to a deeper appreciation of its benefits [8]. Moreover, many course participants were local clinician-educators and some were residency core faculty or program directors. These participants were often sent to the course to learn how to develop and implement POCUS training at their local VA medical center. Although our post-course survey did not collect data on curriculum development, an upcoming national survey of VA medical centers in 2025–2026 will further explore trends in POCUS use and the availability of local training programs.

The benefits of ultrasound-guided procedures can significantly impact large healthcare systems given the increasing number of patients with cirrhosis who require frequent paracenteses [16]. Ultrasound guidance for paracentesis has been previously shown to reduce the risk of bleeding complications by 68% in an observational cohort study. Bleeding complications increase hospitalization costs by $19,066 and length of stay by 4.3 days and are associated with higher mortality [4]. In two single-center observational studies, ultrasound-guided paracentesis performed by trained clinicians had comparable outcomes as interventional radiology-performed procedures and was associated with reduced hospital costs [6, 17]. Further, a recent retrospective study of over 10,000 veteran patients with cirrhosis and ascites found that early paracentesis was associated with lower risk of acute kidney injury, transfer to an intensive care unit, and inpatient mortality; but lack of trained personnel may lead to procedural delays and adverse outcomes [16]. 

We recognize our study has limitations. Inaccuracies in coding may have occurred but should have been balanced between the trained and matched control facilities. We were limited to ICD-10 paracentesis complication codes for bleeding and bowel injury which resulted in very low observed complication rates and reduced power to detect differences in complication rates over time. In addition, the management required for bleeding complications (observation, blood transfusion, embolization, surgery, etc.) is also unknown in our study. Bleeding complications in paracentesis are clinically significant but uncommon, and prior studies have been unable to show significant decreases in bleeding events with POCUS [4, 18]. In a 2018 pre- to post-intervention study of 5,777 paracenteses, the additional use of a high-frequency ultrasound probe to identify prominent blood vessels in the abdominal wall pre-procedure resulted in a lower but statistically insignificant rate of major bleeding [18]. A longitudinal prospective study with a large number of participants performing paracentesis may help to detect differences in complication rates. Ascites leakage, a common complication [2], was not assessed due to lack of a code for this complication, nor were we able to assess for other complications including post-paracentesis circulatory dysfunction and acute kidney injury. The impact of a POCUS training course on these other outcomes warrants further study.

Second, due to inaccuracies in provider-level coding, we analyzed only facility-level coding data but also included provider-reported survey data on frequency of ultrasound usage and complications. We excluded radiology-performed procedures in the analysis, but we could not link specific procedure codes to individual providers or specialties, including those who participated in the training course.

Finally, it is unclear why the matched control facilities had higher usage of ultrasound guidance for paracentesis at baseline than the trained facilities. There were no significant differences in characteristics between the groups in our study, including general POCUS use and availability of local POCUS training, but we cannot rule out other differences which may have impacted the results. One example is availability of dedicated procedure services. Procedure services routinely incorporate ultrasound guidance for procedures and are associated with favorable success and complication rates [19, 20]. Higher prevalence of procedure services in the matched control facilities may explain the baseline findings.

## Conclusions

VA facilities that participated in a national POCUS training course demonstrated an increase in usage of ultrasound guidance for paracentesis, but without significant changes in bleeding complication rates. These results support investment of large healthcare systems in POCUS training to improve adoption of ultrasound guidance for paracentesis– a standard of care practice which could potentially improve outcomes.

## Electronic supplementary material

Below is the link to the electronic supplementary material.

**Supplementary Material 1**: **Additional File 1.** ICD-10 PCS & CPT Procedure Codes + ICD-10 CM Complication Codes

**Supplementary Material 2**: **Additional File 2.** VHA Provider POCUS Survey

**Supplementary Material 3**: **Additional File 3.** Paracentesis Complications and Total Number of Paracenteses during Pre-Intervention, Intervention, and Post-Intervention Periods

**Supplementary Material 4**: **Additional File 4.** Use of Ultrasound Guidance by Radiology for Paracentesis from October 2015 to March 2025

**Supplementary Material 5**: **Additional File 5.** Paracentesis Complications per Coding Data

## Data Availability

The datasets used and analyzed during the current study are available from the corresponding author on reasonable request.

## Declarations

### Disclaimer

The contents of this publication do not represent the views of the U.S. Department of Veterans Affairs or the United States Government.

## Abbreviations

POCUS: Point-of-care ultrasound
VA: Veterans affairs
