# Supplementary material for: Paracentesis complication rates and use of ultrasound: impact of a point-of-care ultrasound training course in the veterans affairs healthcare system
Source: BMC Med Educ. 2025 Aug 12;25:1161. doi: 10.1186/s12909-025-07656-z (PMC12341121; doi:10.1186/s12909-025-07656-z)
Supplement: Supplementary file 1 — Supplementary Material 1: Additional File 1. ICD-10 PCS & CPT Procedure Codes + ICD-10 CM Complication Codes [file 12909_2025_7656_MOESM1_ESM.docx]

**Additional File 1. ICD-10 PCS & CPT Procedure Codes + ICD-10 CM Complication Codes**

| **Procedure: Paracentesis** |
| --- |
| *ICD-10-PCS Codes*:  0W9G30Z Drainage of Peritoneal Cavity with Drainage Device, Percutaneous Approach  0W9G3ZX Drainage of Peritoneal Cavity, Percutaneous Approach, Diagnostic  0W9G3ZZ Drainage of Peritoneal Cavity, Percutaneous Approach  0W9J30Z Drainage of Pelvic Cavity with Drainage Device, Percutaneous Approach  0W9J3ZX Drainage of Pelvic Cavity, Percutaneous Approach, Diagnostic  0W9J3ZZ Drainage of Pelvic Cavity, Percutaneous Approach  *CPT Codes*:  CPT Code CPT Description  49082 Abdominal paracentesis (diagnostic or therapeutic); without imaging guidance  49083 Abdominal paracentesis (diagnostic or therapeutic); with imaging guidance  *Considered but not used:*  49084 Peritoneal lavage, including imaging guidance, when performed |
| **Complications: Paracentesis** |
| *ICD-10-CM Codes (when diagnosis occurs at an outpatient visit within 5 days (120 hrs) of one of the above procedures, or a hospitalization with an admit date/time within 120 hrs of one of the above procedures, or during a hospitalization which also includes one of the above procedures):*  K66.1 Hemoperitoneum  S36.400A Unspecified injury of duodenum, initial encounter  S36.408A Unspecified injury of other part of small intestine, initial encounter  S36.409A Unspecified injury of unspecified part of small intestine, initial encounter  S36.490A Other injury of duodenum, initial encounter  S36.498A Other injury of other part of small intestine, initial encounter  S36.499A Other injury of unspecified part of small intestine, initial encounter  S36.500A Unspecified injury of ascending [right] colon, initial encounter  S36.501A Unspecified injury of transverse colon, initial encounter  S36.502A Unspecified injury of descending [left] colon, initial encounter  S36.508A Unspecified injury of other part of colon, initial encounter  S36.509A Unspecified injury of unspecified part of colon, initial encounter  S36.590A Other injury of ascending [right] colon, initial encounter  S36.591A Other injury of transverse colon, initial encounter  S36.592A Other injury of descending [left] colon, initial encounter  S36.598A Other injury of other part of colon, initial encounter  S36.599A Other injury of unspecified part of colon, initial encounter  *Codes considered but not used:*  K92.2 Gastrointestinal haemorrhage, unspecified  S36.400D Unspecified injury of duodenum, subsequent encounter  S36.400S Unspecified injury of duodenum, sequela  S36.408D Unspecified injury of other part of small intestine, subsequent encounter  S36.408S Unspecified injury of other part of small intestine, sequela  S36.409D Unspecified injury of unspecified part of small intestine, subsequent encounter  S36.409S Unspecified injury of unspecified part of small intestine, sequela  S36.490D Other injury of duodenum, subsequent encounter  S36.490S Other injury of duodenum, sequela  S36.498D Other injury of other part of small intestine, subsequent encounter  S36.498S Other injury of other part of small intestine, sequela  S36.499D Other injury of unspecified part of small intestine, subsequent encounter  S36.499S Other injury of unspecified part of small intestine, sequela  S36.500D Unspecified injury of ascending [right] colon, subsequent encounter  S36.500S Unspecified injury of ascending [right] colon, sequela  S36.501D Unspecified injury of transverse colon, subsequent encounter  S36.501S Unspecified injury of transverse colon, sequela  S36.502D Unspecified injury of descending [left] colon, subsequent encounter  S36.502S Unspecified injury of descending [left] colon, sequela  S36.508D Unspecified injury of other part of colon, subsequent encounter  S36.508S Unspecified injury of other part of colon, sequela  S36.509D Unspecified injury of unspecified part of colon, subsequent encounter  S36.509S Unspecified injury of unspecified part of colon, sequela  S36.590D Other injury of ascending [right] colon, subsequent encounter  S36.590S Other injury of ascending [right] colon, sequela  S36.591D Other injury of transverse colon, subsequent encounter  S36.591S Other injury of transverse colon, sequela  S36.592D Other injury of descending [left] colon, subsequent encounter  S36.592S Other injury of descending [left] colon, sequela  S36.598D Other injury of other part of colon, subsequent encounter  S36.598S Other injury of other part of colon, sequela  S36.599D Other injury of unspecified part of colon, subsequent encounter  S36.599S Other injury of unspecified part of colon, sequela |

ICD-10 PCS, International Classification of Diseases 10^th^ Revision Procedure Coding System; CPT, Current Procedural Terminology; ICD-10 CM, International Classification of Diseases 10^th^ Revision Clinical Modification.
