# Supplementary material for: Paracentesis complication rates and use of ultrasound: impact of a point-of-care ultrasound training course in the veterans affairs healthcare system
Source: BMC Med Educ. 2025 Aug 12;25:1161. doi: 10.1186/s12909-025-07656-z (PMC12341121; doi:10.1186/s12909-025-07656-z)
Supplement: Supplementary file 2 — Supplementary Material 2: Additional File 2. VHA Provider POCUS Survey [file 12909_2025_7656_MOESM2_ESM.pdf]

VHA Provider POCUS Survey

VHA National Point-of-care Ultrasound Training Program

Point of Care Ultrasound (POCUS) definition: “Point-of-care ultrasound is defined as a goal-directed ultrasound examination performed by a healthcare provider at the bedside to answer a specific diagnostic question in real-time or guide performance of an invasive procedure”

The purpose of this survey is to assess current use of Point of Care Ultrasound (POCUS) by providers in acute care settings in the VHA

Each provider participating in the POCUS training course at the SimLEARN national simulation center will be surveyed pre-course and 4-6 months post-course. This survey takes approximately 15 minutes to complete.

Please provide the following provider and facility information.

1) Last name of provider completing survey:

____

2) First name of provider:

____

3) Email:

____

4) Phone Number / Extension:

____

6) Age:

◯ 20 ◯ 21 ◯ 22 ◯ 23 ◯ 24 ◯ 25 ◯ 26 ◯ 27 ◯ 28 ◯ 29 ◯ 30 ◯ 31
◯ 32 ◯ 33 ◯ 34 ◯ 35 ◯ 36 ◯ 37 ◯ 38 ◯ 39 ◯ 40 ◯ 41 ◯ 42 ◯ 43
◯ 44 ◯ 45 ◯ 46 ◯ 47 ◯ 48 ◯ 49 ◯ 50 ◯ 51 ◯ 52 ◯ 53 ◯ 54 ◯ 55
◯ 56 ◯ 57 ◯ 58 ◯ 59 ◯ 60 ◯ 61 ◯ 62 ◯ 63 ◯ 64 ◯ 65 ◯ 66 ◯ 67
◯ 68 ◯ 69 ◯ 70 ◯ 71 ◯ 72 ◯ 73 ◯ 74 ◯ 75 ◯ 76 ◯ 77 ◯ 78 ◯ 79
◯ 80 ◯ > 81

5) Gender:

◯ Female ◯ Male

7) Years in practice since completion of residency / fellowship:

◯ 1 ◯ 2 ◯ 3 ◯ 4 ◯ 5 ◯ 6 ◯ 7 ◯ 8 ◯ 9 ◯ 10 ◯ 11 ◯ 12 ◯ 13
◯ 14 ◯ 15 ◯ 16 ◯ 17 ◯ 18 ◯ 19 ◯ 20 ◯ 21 ◯ 22 ◯ 23 ◯ 24 ◯ 25
◯ 26 ◯ 27 ◯ 28 ◯ 29 ◯ 30 ◯ 31 ◯ 32 ◯ 33 ◯ 34 ◯ 35 ◯ 36 ◯ 37
◯ 38 ◯ 39 ◯ 40 ◯ 41 ◯ 42 ◯ 43 ◯ 44 ◯ 45 ◯ 46 ◯ 47 ◯ 48 ◯ 49
◯ 50 ◯ 51 ◯ 52 ◯ 53 ◯ 54 ◯ 55 ◯ >55

8) What do you consider your Primary Specialty:

◯ Emergency Medicine ◯ Internal Medicine ◯ Critical Care ◯ Pulmonary ◯ Rheumatology
◯ Endocrinology ◯ Nephrology ◯ Gastroenterology ◯ Anesthesiology ◯ Orthopedic Surgery
◯ Vascular Surgery ◯ General Surgery ◯ Trauma Surgery ◯ Urology ◯ Ophthalmology
◯ Other

Please specify:

____

9) During an average week, what percentage of time do you spend in the following activities:

a. Direct patient care

◯ 0% ◯ 5% ◯ 10% ◯ 15% ◯ 20% ◯ 25% ◯ 30% ◯ 35% ◯ 40% ◯ 45%
◯ 50% ◯ 55% ◯ 60% ◯ 65% ◯ 70% ◯ 75% ◯ 80% ◯ 85% ◯ 90% ◯ 95%
◯ 100%

b. Supervising Patient Care by Trainees

◯ 0% ◯ 5% ◯ 10% ◯ 15% ◯ 20% ◯ 25% ◯ 30% ◯ 35% ◯ 40% ◯ 45%
◯ 50% ◯ 55% ◯ 60% ◯ 65% ◯ 70% ◯ 75% ◯ 80% ◯ 85% ◯ 90% ◯ 95%
◯ 100%

c. Non-clinical activities (administration, research)

◯ 0% ◯ 5% ◯ 10% ◯ 15% ◯ 20% ◯ 25% ◯ 30% ◯ 35% ◯ 40% ◯ 45%
◯ 50% ◯ 55% ◯ 60% ◯ 65% ◯ 70% ◯ 75% ◯ 80% ◯ 85% ◯ 90% ◯ 95%
◯ 100%

Total time on work activities

____
(Please ensure total equal 100%)

10) Facility Number and Name
(Facilities are listed by state starting with Alaska)

◯ 463 Alaska VA Healthcare System ◯ 521 Birmingham VA Medical Center ◯ 619A4 Central Alabama Veterans Health Care System East Campus ◯ 619 Central Alabama Veterans Health Care System West Campus ◯ 679 Tuscaloosa VA Medical Center ◯ 598 Central Arkansas Veterans Healthcare System Eugene J. Towbin Healthcare Center ◯ 598 Central Arkansas Veterans Healthcare System John L. McClellan Memorial Veterans Hospital ◯ 564 Veterans Health Care System of the Ozarks ◯ 649 Northern Arizona VA Health Care System ◯ 644 Phoenix VA Health Care System ◯ 678 Southern Arizona VA Health Care System ◯ 570 Central California VA Health Care System ◯ 640 Livermore ◯ 640 Menlo Park ◯ 662 San Francisco VA Health Care System ◯ 691 VA Greater Los Angeles Healthcare System (GLA) ◯ 605 VA Loma Linda Healthcare System ◯ 600 VA Long Beach Healthcare System ◯ 612 VA Northern California Health Care System ◯ 640 VA Palo Alto Health Care System ◯ 664 VA San Diego Healthcare System ◯ 575 Grand Junction VA Medical Center ◯ 554 VA Eastern Colorado Health Care System(ECHCS) ◯ 689 VA Connecticut Healthcare System ◯ 689A4 VA Connecticut Healthcare System, Newington Campus ◯ 689 VA Connecticut Healthcare System, West Haven Campus ◯ 688 Washington DC VA Medical Center ◯ 460 Wilmington VA Medical Center ◯ 516 Bay Pines VA Healthcare System ◯ 673 James A. Haley Veterans' Hospital ◯ 673 James A. Haley Veterans' Hospital Primary Care Annex ◯ 573A4 Lake City VAMC, NF/SGVHS ◯ 573 Malcom Randall VAMC, NF/SGVHS ◯ 546 Miami VA Healthcare System ◯ 573 North Florida/South Georgia Veterans Health System ◯ 675 Orlando VA Medical Center ◯ 548 West Palm Beach VAMC ◯ 508 Atlanta VA Health Care System ◯ 557 Carl Vinson VA Medical Center ◯ 509 Charlie Norwood VA Medical Center ◯ 459 VA Pacific Islands Health Care System ◯ 636A8 Iowa City VA Health Care System ◯ 636A6 VA Central Iowa Health Care System ◯ 531 Boise VA Medical Center ◯ 556 Captain James A. Lovell Federal Health Care Center ◯ 578 Edward Hines Jr. VA Hospital ◯ 537 Jesse Brown VA Medical Center ◯ 657A5 Marion VA Medical Center ◯ 550 VA Illiana Health Care System ◯ 583 Richard L. Roudebush VA Medical Center (Indianapolis VA Medical Center) ◯ 610 VA Northern Indiana Health Care System ◯ 610 "VA Northern Indiana Health Care System ◯ - Marion Campus" ◯ 610A4 VA Northern Indiana Health Care System-Fort Wayne Campus ◯ 589A7 Robert J. Dole VA Medical Center ◯ 589A5 VA Eastern Kansas Health Care System - Colmery-O'Neil VA Medical Center ◯ 589A6 VA Eastern Kansas Health Care System - Dwight D. Eisenhower VA Medical Center ◯ 596 Lexington VA Medical Center ◯ 596A4 Lexington VAMC: Cooper Division ◯ 596 Lexington VAMC: Leestown Division ◯ 603 Robley Rex VA Medical Center ◯ 502 Alexandria VA Health Care System ◯ 667 Overton Brooks VA Medical Center ◯ 629 Southeast Louisiana Veterans Health Care System ◯ 518 Edith Nourse Rogers Memorial Veterans Hospital (Bedford VA) ◯ 523 VA Boston Healthcare System ◯ 523A5 VA Boston Healthcare System, Brockton Campus ◯ 523 VA Boston Healthcare System, Jamaica Plain Campus ◯ 523A4 VA Boston Healthcare System, West Roxbury Campus ◯ 631 VA Central Western Massachusetts Healthcare System ◯ 512 Baltimore VA Medical Center - VA Maryland Health Care System ◯ 512GB Loch Raven VA Community Living & Rehabilitation Center ◯ 512A5 Perry Point VA Medical Center - VA Maryland Health Care System ◯ 512 VA Maryland Health Care System ◯ 402 VA Maine Healthcare System - Togus ◯ 655 Aleda E. Lutz VA Medical Center ◯ 515 Battle Creek VA Medical Center ◯ 553 John D. Dingell VA Medical Center ◯ 585 Oscar G. Johnson VA Medical Center ◯ 506 VA Ann Arbor Healthcare System ◯ 618 Minneapolis VA Health Care System ◯ 656 St. Cloud VA Health Care System ◯ 589A4 Harry S. Truman Memorial ◯ 657A4 John J. Pershing VA Medical Center ◯ 589 Kansas City VA Medical Center ◯ 657 VA St. Louis Health Care System ◯ 657A0 VA St. Louis Health Care System - Jefferson Barracks Division ◯ 657 VA St. Louis Health Care System - John Cochran Division ◯ 586 G.V. (Sonny) Montgomery VA Medical Center ◯ 520 Gulf Coast Veterans Health Care System ◯ 436 VA Montana Health Care System ◯ 637 Asheville VA Medical Center ◯ 558 Durham VA Medical Center ◯ 565 Fayetteville VA Medical Center ◯ 659 Salisbury - W.G. (Bill) Hefner VA Medical Center ◯ 437 Fargo VA Health Care System ◯ 636A4 Grand Island VA Medical Center ◯ 636 Omaha VA Medical Center--VA Nebraska-Western Iowa HCS ◯ 608 Manchester VA Medical Center ◯ 561 East Orange Campus of the VA New Jersey Health Care System ◯ 561A4 Lyons Campus of the VA New Jersey Health Care System ◯ 561 VA New Jersey Health Care System ◯ 501 New Mexico VA Health Care System ◯ 654 VA Sierra Nevada Health Care System ◯ 593 VA Southern Nevada Healthcare System ◯ 528A8 Albany VA Medical Center: Samuel S. Stratton ◯ 528A6 Bath VA Medical Center ◯ 630A4 Brooklyn Campus of the VA NY Harbor Healthcare System ◯ 528A5 Canandaigua VA Medical Center ◯ 620A4 Castle Point Campus of the VA Hudson Valley Health Care System ◯ 620 Franklin Delano Roosevelt Campus of the VA Hudson Valley Health Care System (Montrose) ◯ 526 James J. Peters VA Medical Center (Bronx, NY) ◯ 630 Manhattan Campus of the VA NY Harbor Healthcare System ◯ 632 Northport VA Medical Center ◯ 528A7 Syracuse VA Medical Center ◯ 620 VA Hudson Valley Health Care System ◯ 630 VA NY Harbor Healthcare System ◯ 528 VA Western New York Healthcare System ◯ 528A4 VA Western New York Healthcare System at Batavia ◯ 528 VA Western New York Healthcare System at Buffalo ◯ 757 Chalmers P. Wylie Ambulatory Care Center ◯ 538 Chillicothe VA Medical Center ◯ 539 Cincinnati VA Medical Center ◯ 552 Dayton VA Medical Center ◯ 541 Louis Stokes Cleveland VA Medical Center ◯ 717 Eastern Oklahoma VA Health Care System (Jack C. Montgomery VAMC) ◯ 635 Oklahoma City VA Health Care System ◯ 648 VA Portland Health Care System ◯ 653 VA Roseburg Healthcare System ◯ 692 White City or VA Southern Oregon Rehabilitation Center ◯ 503 Altoona - James E. Van Zandt VA Medical Center ◯ 542 Coatesville VA Medical Center ◯ 562 Erie VA Medical Center ◯ 595 Lebanon VA Medical Center ◯ 642 Philadelphia VA Medical Center ◯ 529 VA Butler Healthcare ◯ 646 VA Pittsburgh Healthcare System ◯ 646A4 VA Pittsburgh Healthcare System, H.J. Heinz Campus ◯ 646 VA Pittsburgh Healthcare System, University Drive Campus ◯ 693 Wilkes-Barre VA Medical Center

◯ 672 VA Caribbean Healthcare System ◯ 650 Providence VA Medical Center ◯ 534 Ralph H. Johnson VA Medical Center ◯ 544 Wm. Jennings Bryan Dorn VA Medical Center ◯ 438 Royal C. Johnson Veterans Memorial Medical Center ◯ 568A4 VA Black Hills Health Care System - Hot Springs Campus ◯ 568 VA Black Hills Health Care System - Fort Meade Campus ◯ 614 Memphis VA Medical Center ◯ 621 Mountain Home VAMC/Johnson City ◯ 626 Tennessee Valley Healthcare System ◯ 626A4 Tennessee Valley Healthcare System - Alvin C. York (Murfreesboro) Campus ◯ 626 Tennessee Valley Healthcare System - Nashville Campus ◯ 504 Amarillo VA Health Care System ◯ 674 Central Texas Veterans Health Care System ◯ 549 Dallas VA Medical Center ◯ 674A4 Doris Miller Department of Veterans Affairs Medical Center ◯ 756 El Paso VA Health Care System ◯ 671A4 Kerrville VA Hospital ◯ 580 Michael E. DeBakey VA Medical Center ◯ 549A4 Sam Rayburn Memorial Veterans Center ◯ 671 South Texas Veterans Health Care System ◯ 740 VA Health Care Center at Harlingen ◯ 549 VA North Texas Health Care System ◯ 740 VA Texas Valley Coastal Bend Health Care System ◯ 519 West Texas VA Health Care System ◯ 660 VA Salt Lake City Health Care System ◯ 590 Hampton VA Medical Center ◯ 652 Hunter Holmes McGuire VA Medical Center ◯ 658 Salem VA Medical Center ◯ 405 White River Junction VA Medical Center ◯ 687 Jonathan M. Wainwright Memorial VA Medical Center ◯ 668 Mann-Grandstaff VA Medical Center ◯ 648A4 VA Portland Health Care System - Vancouver Campus ◯ 663 VA Puget Sound Health Care System ◯ 663A4 VA Puget Sound Health Care System - American Lake Division ◯ 663 VA Puget Sound Health Care System - Seattle Division ◯ 695 Clement J. Zablocki Veterans Affairs Medical Center ◯ 676 Tomah VA Medical Center ◯ 607 William S. Middleton Memorial Veterans Hospital ◯ 517 Beckley VA Medical Center ◯ 540 Clarksburg - Louis A. Johnson VA Medical Center ◯ 581 Huntington VA Medical Center ◯ 613 Martinsburg VA Medical Center ◯ 442 Cheyenne VA Medical ◯ 666 Sheridan VA Medical Center

POCUS Cardiac Applications

Have you used POCUS for CARDIAC applications when caring for patients in the past 6 months?

◯ Yes ◯ No

On average, how many times per week do you use POCUS for each of the applications listed below:

a. Left Ventricular Systolic Function

◯ 0 ◯ 1 ◯ 2 ◯ 3 ◯ 4 ◯ 5 ◯ 6 ◯ 7 ◯ 8 ◯ 9 ◯ 10 ◯ 11 ◯ 12
◯ 13 ◯ 14 ◯ 15 ◯ 16 ◯ 17 ◯ 18 ◯ 19 ◯ 20 ◯ 21 ◯ 22 ◯ 23 ◯ 24
◯ 25 ◯ 26 ◯ 27 ◯ 28 ◯ 29 ◯ 30 ◯ >30

b. Volume Status [inferior vena cava (IVC)/ internal jugular vein (IJ)]

◯ 0 ◯ 1 ◯ 2 ◯ 3 ◯ 4 ◯ 5 ◯ 6 ◯ 7 ◯ 8 ◯ 9 ◯ 10 ◯ 11 ◯ 12
◯ 13 ◯ 14 ◯ 15 ◯ 16 ◯ 17 ◯ 18 ◯ 19 ◯ 20 ◯ 21 ◯ 22 ◯ 23 ◯ 24
◯ 25 ◯ 26 ◯ 27 ◯ 28 ◯ 29 ◯ 30 ◯ >30

c. Advanced Hemodynamics (e.g., cardiac output, stroke volume)

◯ 0 ◯ 1 ◯ 2 ◯ 3 ◯ 4 ◯ 5 ◯ 6 ◯ 7 ◯ 8 ◯ 9 ◯ 10 ◯ 11 ◯ 12
◯ 13 ◯ 14 ◯ 15 ◯ 16 ◯ 17 ◯ 18 ◯ 19 ◯ 20 ◯ 21 ◯ 22 ◯ 23 ◯ 24
◯ 25 ◯ 26 ◯ 27 ◯ 28 ◯ 29 ◯ 30 ◯ >30

d. Pericardial Effusion

○ 0 ○ 1 ○ 2 ○ 3 ○ 4 ○ 5 ○ 6 ○ 7 ○ 8 ○ 9 ○ 10 ○ 11 ○ 12
○ 13 ○ 14 ○ 15 ○ 16 ○ 17 ○ 18 ○ 19 ○ 20 ○ 21 ○ 22 ○ 23 ○ 24
○ 25 ○ 26 ○ 27 ○ 28 ○ 29 ○ 30 ○ >30

e. Pericardiocentesis

○ 0 ○ 1 ○ 2 ○ 3 ○ 4 ○ 5 ○ 6 ○ 7 ○ 8 ○ 9 ○ 10 ○ 11 ○ 12
○ 13 ○ 14 ○ 15 ○ 16 ○ 17 ○ 18 ○ 19 ○ 20 ○ 21 ○ 22 ○ 23 ○ 24
○ 25 ○ 26 ○ 27 ○ 28 ○ 29 ○ 30 ○ >30

Please rank how comfortable you are with the following applications:

a. Left Ventricular Systolic Function

not comfortable at all | somewhat comfortable | very comfortable

(Place a mark on the scale above)

b. Volume Status [inferior vena cava (IVC)/ internat jugular vein (IJ)]

not comfortable at all | somewhat comfortable | very comfortable

(Place a mark on the scale above)

c. Advanced Hemodynamics (e.g., cardiac output, stroke volume)

not comfortable at all | somewhat comfortable | very comfortable

(Place a mark on the scale above)

d. Pericardial Effusion

not comfortable at all | somewhat comfortable | very comfortable

(Place a mark on the scale above)

e. Pericardiocentesis

not comfortable at all | somewhat comfortable | very comfortable

(Place a mark on the scale above)

POCUS Pulmonary Applications

Have you used POCUS for PULMONARY applications when caring for patients in the past 6 months?

◯ Yes ◯ No

On average, how many times per week do you use POCUS for each of the applications listed below:

a. Pleural Effusion

◯ 0 ◯ 1 ◯ 2 ◯ 3 ◯ 4 ◯ 5 ◯ 6 ◯ 7 ◯ 8 ◯ 9 ◯ 10 ◯ 11 ◯ 12
◯ 13 ◯ 14 ◯ 15 ◯ 16 ◯ 17 ◯ 18 ◯ 19 ◯ 20 ◯ 21 ◯ 22 ◯ 23 ◯ 24
◯ 25 ◯ 26 ◯ 27 ◯ 28 ◯ 29 ◯ 30 ◯ >30

b. Pneumothorax

◯ 0 ◯ 1 ◯ 2 ◯ 3 ◯ 4 ◯ 5 ◯ 6 ◯ 7 ◯ 8 ◯ 9 ◯ 10 ◯ 11 ◯ 12
◯ 13 ◯ 14 ◯ 15 ◯ 16 ◯ 17 ◯ 18 ◯ 19 ◯ 20 ◯ 21 ◯ 22 ◯ 23 ◯ 24
◯ 25 ◯ 26 ◯ 27 ◯ 28 ◯ 29 ◯ 30 ◯ >30

c. Pulmonary Edema

◯ 0 ◯ 1 ◯ 2 ◯ 3 ◯ 4 ◯ 5 ◯ 6 ◯ 7 ◯ 8 ◯ 9 ◯ 10 ◯ 11 ◯ 12
◯ 13 ◯ 14 ◯ 15 ◯ 16 ◯ 17 ◯ 18 ◯ 19 ◯ 20 ◯ 21 ◯ 22 ◯ 23 ◯ 24
◯ 25 ◯ 26 ◯ 27 ◯ 28 ◯ 29 ◯ 30 ◯ >30

d. Pneumonia

◯ 0 ◯ 1 ◯ 2 ◯ 3 ◯ 4 ◯ 5 ◯ 6 ◯ 7 ◯ 8 ◯ 9 ◯ 10 ◯ 11 ◯ 12
◯ 13 ◯ 14 ◯ 15 ◯ 16 ◯ 17 ◯ 18 ◯ 19 ◯ 20 ◯ 21 ◯ 22 ◯ 23 ◯ 24
◯ 25 ◯ 26 ◯ 27 ◯ 28 ◯ 29 ◯ 30 ◯ >30

e. Thoracentesis

◯ 0 ◯ 1 ◯ 2 ◯ 3 ◯ 4 ◯ 5 ◯ 6 ◯ 7 ◯ 8 ◯ 9 ◯ 10 ◯ 11 ◯ 12
◯ 13 ◯ 14 ◯ 15 ◯ 16 ◯ 17 ◯ 18 ◯ 19 ◯ 20 ◯ 21 ◯ 22 ◯ 23 ◯ 24
◯ 25 ◯ 26 ◯ 27 ◯ 28 ◯ 29 ◯ 30 ◯ >30

f. Chest tube insertion

◯ 0 ◯ 1 ◯ 2 ◯ 3 ◯ 4 ◯ 5 ◯ 6 ◯ 7 ◯ 8 ◯ 9 ◯ 10 ◯ 11 ◯ 12
◯ 13 ◯ 14 ◯ 15 ◯ 16 ◯ 17 ◯ 18 ◯ 19 ◯ 20 ◯ 21 ◯ 22 ◯ 23 ◯ 24
◯ 25 ◯ 26 ◯ 27 ◯ 28 ◯ 29 ◯ 30 ◯ >30

Please rank how comfortable you are with the following applications:

a. Pleural Effusion

not comfortable at all — somewhat comfortable — very comfortable

(Place a mark on the scale above)

b. Pneumothorax

not comfortable at all — somewhat comfortable — very comfortable

(Place a mark on the scale above)

c. Pulmonary Edema

not comfortable at all — somewhat comfortable — very comfortable

(Place a mark on the scale above)

d. Pneumonia

not comfortable at all — somewhat comfortable — very comfortable

(Place a mark on the scale above)

e. Thoracentesis

not comfortable at all — somewhat comfortable — very comfortable

(Place a mark on the scale above)

f. Chest tube insertion

not comfortable at all — somewhat comfortable — very comfortable

(Place a mark on the scale above)

POCUS Gastrointestinal Applications

Have you used POCUS for GASTROINTESTINAL applications when caring for patients in the past 6 months?

◯ Yes ◯ No

On average, how many times per week do you use POCUS for each of the applications listed below:

a.Biliary

◯ 0 ◯ 1 ◯ 2 ◯ 3 ◯ 4 ◯ 5 ◯ 6 ◯ 7 ◯ 8 ◯ 9 ◯ 10 ◯ 11 ◯ 12
◯ 13 ◯ 14 ◯ 15 ◯ 16 ◯ 17 ◯ 18 ◯ 19 ◯ 20 ◯ 21 ◯ 22 ◯ 23 ◯ 24
◯ 25 ◯ 26 ◯ 27 ◯ 28 ◯ 29 ◯ 30 ◯ >30

b. Peritoneal Fluid

◯ 0 ◯ 1 ◯ 2 ◯ 3 ◯ 4 ◯ 5 ◯ 6 ◯ 7 ◯ 8 ◯ 9 ◯ 10 ◯ 11 ◯ 12
◯ 13 ◯ 14 ◯ 15 ◯ 16 ◯ 17 ◯ 18 ◯ 19 ◯ 20 ◯ 21 ◯ 22 ◯ 23 ◯ 24
◯ 25 ◯ 26 ◯ 27 ◯ 28 ◯ 29 ◯ 30 ◯ >30

c. Hernia

◯ 0 ◯ 1 ◯ 2 ◯ 3 ◯ 4 ◯ 5 ◯ 6 ◯ 7 ◯ 8 ◯ 9 ◯ 10 ◯ 11 ◯ 12
◯ 13 ◯ 14 ◯ 15 ◯ 16 ◯ 17 ◯ 18 ◯ 19 ◯ 20 ◯ 21 ◯ 22 ◯ 23 ◯ 24
◯ 25 ◯ 26 ◯ 27 ◯ 28 ◯ 29 ◯ 30 ◯ >30

d. Paracentesis

○ 0 ○ 1 ○ 2 ○ 3 ○ 4 ○ 5 ○ 6 ○ 7 ○ 8 ○ 9 ○ 10 ○ 11 ○ 12
○ 13 ○ 14 ○ 15 ○ 16 ○ 17 ○ 18 ○ 19 ○ 20 ○ 21 ○ 22 ○ 23 ○ 24
○ 25 ○ 26 ○ 27 ○ 28 ○ 29 ○ 30 ○ >30

Please rank how comfortable you are with the following procedures:

a. Biliary

not comfortable at all | somewhat comfortable | very comfortable

(Place a mark on the scale above)

b. Peritoneal Fluid

not comfortable at all | somewhat comfortable | very comfortable

(Place a mark on the scale above)

c. Hernia

not comfortable at all | somewhat comfortable | very comfortable

(Place a mark on the scale above)

d. Paracentesis

not comfortable at all | somewhat comfortable | very comfortable

(Place a mark on the scale above)

POCUS Urinary Applications

Have you used POCUS for URINARY applications when caring for patients in the past 6 months?

◯ Yes ◯ No

On average, how many times per week do you use POCUS for each of the applications listed below:

a. Hydronephrosis

◯ 0 ◯ 1 ◯ 2 ◯ 3 ◯ 4 ◯ 5 ◯ 6 ◯ 7 ◯ 8 ◯ 9 ◯ 10 ◯ 11 ◯ 12
◯ 13 ◯ 14 ◯ 15 ◯ 16 ◯ 17 ◯ 18 ◯ 19 ◯ 20 ◯ 21 ◯ 22 ◯ 23 ◯ 24
◯ 25 ◯ 26 ◯ 27 ◯ 28 ◯ 29 ◯ 30 ◯ >30

b. Nephrolithiasis

◯ 0 ◯ 1 ◯ 2 ◯ 3 ◯ 4 ◯ 5 ◯ 6 ◯ 7 ◯ 8 ◯ 9 ◯ 10 ◯ 11 ◯ 12
◯ 13 ◯ 14 ◯ 15 ◯ 16 ◯ 17 ◯ 18 ◯ 19 ◯ 20 ◯ 21 ◯ 22 ◯ 23 ◯ 24
◯ 25 ◯ 26 ◯ 27 ◯ 28 ◯ 29 ◯ 30 ◯ >30

c. Urinary Retention

◯ 0 ◯ 1 ◯ 2 ◯ 3 ◯ 4 ◯ 5 ◯ 6 ◯ 7 ◯ 8 ◯ 9 ◯ 10 ◯ 11 ◯ 12
◯ 13 ◯ 14 ◯ 15 ◯ 16 ◯ 17 ◯ 18 ◯ 19 ◯ 20 ◯ 21 ◯ 22 ◯ 23 ◯ 24
◯ 25 ◯ 26 ◯ 27 ◯ 28 ◯ 29 ◯ 30 ◯ >30

d. Suprapubic Catheter Insertion

○ 0 ○ 1 ○ 2 ○ 3 ○ 4 ○ 5 ○ 6 ○ 7 ○ 8 ○ 9 ○ 10 ○ 11 ○ 12
○ 13 ○ 14 ○ 15 ○ 16 ○ 17 ○ 18 ○ 19 ○ 20 ○ 21 ○ 22 ○ 23 ○ 24
○ 25 ○ 26 ○ 27 ○ 28 ○ 29 ○ 30 ○ >30

Please rank how comfortable you are with the following procedures:

a. Hydronephrosis

not comfortable at all	somewhat comfortable	very comfortable

(Place a mark on the scale above)

b. Nephrolithiasis

not comfortable at all	somewhat comfortable	very comfortable

(Place a mark on the scale above)

c. Urinary Retention

not comfortable at all	somewhat comfortable	very comfortable

(Place a mark on the scale above)

d. Suprapubic Catheter Insertion

not comfortable at all	somewhat comfortable	very comfortable

(Place a mark on the scale above)

POCUS Gynecological Applications

Have you used POCUS for GYNECOLOGICAL applications when caring for patients in the past 6 months?

○ Yes ○ No

On average, how many times per week do you use POCUS for each of the applications listed below:

a. Intrauterine Pregnancy

○ 0 ○ 1 ○ 2 ○ 3 ○ 4 ○ 5 ○ 6 ○ 7 ○ 8 ○ 9 ○ 10 ○ 11 ○ 12
○ 13 ○ 14 ○ 15 ○ 16 ○ 17 ○ 18 ○ 19 ○ 20 ○ 21 ○ 22 ○ 23 ○ 24
○ 25 ○ 26 ○ 27 ○ 28 ○ 29 ○ 30 ○ >30

Please rank how comfortable you are with the following procedures:

a. Intrauterine Pregnancy

not comfortable at all — somewhat comfortable — very comfortable

(Place a mark on the scale above)

POCUS Vascular Applications

Have you used POCUS for VASCULAR applications when caring for patients in the past 6 months?

◯ Yes ◯ No

On average, how many times per week do you use POCUS for each of the applications listed below:

a. Abdominal Aortic Aneurism (AAA)

◯ 0 ◯ 1 ◯ 2 ◯ 3 ◯ 4 ◯ 5 ◯ 6 ◯ 7 ◯ 8 ◯ 9 ◯ 10 ◯ 11 ◯ 12
◯ 13 ◯ 14 ◯ 15 ◯ 16 ◯ 17 ◯ 18 ◯ 19 ◯ 20 ◯ 21 ◯ 22 ◯ 23 ◯ 24
◯ 25 ◯ 26 ◯ 27 ◯ 28 ◯ 29 ◯ 30 ◯ >30

b. Deep Vein Thrombosis (DVT)

◯ 0 ◯ 1 ◯ 2 ◯ 3 ◯ 4 ◯ 5 ◯ 6 ◯ 7 ◯ 8 ◯ 9 ◯ 10 ◯ 11 ◯ 12
◯ 13 ◯ 14 ◯ 15 ◯ 16 ◯ 17 ◯ 18 ◯ 19 ◯ 20 ◯ 21 ◯ 22 ◯ 23 ◯ 24
◯ 25 ◯ 26 ◯ 27 ◯ 28 ◯ 29 ◯ 30 ◯ >30

c. Arterial Flow

◯ 0 ◯ 1 ◯ 2 ◯ 3 ◯ 4 ◯ 5 ◯ 6 ◯ 7 ◯ 8 ◯ 9 ◯ 10 ◯ 11 ◯ 12
◯ 13 ◯ 14 ◯ 15 ◯ 16 ◯ 17 ◯ 18 ◯ 19 ◯ 20 ◯ 21 ◯ 22 ◯ 23 ◯ 24
◯ 25 ◯ 26 ◯ 27 ◯ 28 ◯ 29 ◯ 30 ◯ >30

d. Peripheral IV access Insertion

◯ 0 ◯ 1 ◯ 2 ◯ 3 ◯ 4 ◯ 5 ◯ 6 ◯ 7 ◯ 8 ◯ 9 ◯ 10 ◯ 11 ◯ 12
◯ 13 ◯ 14 ◯ 15 ◯ 16 ◯ 17 ◯ 18 ◯ 19 ◯ 20 ◯ 21 ◯ 22 ◯ 23 ◯ 24
◯ 25 ◯ 26 ◯ 27 ◯ 28 ◯ 29 ◯ 30 ◯ >30

e. Internal Jugular Central Line Insertion

◯ 0 ◯ 1 ◯ 2 ◯ 3 ◯ 4 ◯ 5 ◯ 6 ◯ 7 ◯ 8 ◯ 9 ◯ 10 ◯ 11 ◯ 12
◯ 13 ◯ 14 ◯ 15 ◯ 16 ◯ 17 ◯ 18 ◯ 19 ◯ 20 ◯ 21 ◯ 22 ◯ 23 ◯ 24
◯ 25 ◯ 26 ◯ 27 ◯ 28 ◯ 29 ◯ 30 ◯ >30

f. Subclavian Central Line Insertion

◯ 0 ◯ 1 ◯ 2 ◯ 3 ◯ 4 ◯ 5 ◯ 6 ◯ 7 ◯ 8 ◯ 9 ◯ 10 ◯ 11 ◯ 12
◯ 13 ◯ 14 ◯ 15 ◯ 16 ◯ 17 ◯ 18 ◯ 19 ◯ 20 ◯ 21 ◯ 22 ◯ 23 ◯ 24
◯ 25 ◯ 26 ◯ 27 ◯ 28 ◯ 29 ◯ 30 ◯ >30

g. Femoral Central Line Insertion

◯ 0 ◯ 1 ◯ 2 ◯ 3 ◯ 4 ◯ 5 ◯ 6 ◯ 7 ◯ 8 ◯ 9 ◯ 10 ◯ 11 ◯ 12
◯ 13 ◯ 14 ◯ 15 ◯ 16 ◯ 17 ◯ 18 ◯ 19 ◯ 20 ◯ 21 ◯ 22 ◯ 23 ◯ 24
◯ 25 ◯ 26 ◯ 27 ◯ 28 ◯ 29 ◯ 30 ◯ >30

h. Peripherally Inserted Central Catheter (PICC) Insertion*

◯ 0 ◯ 1 ◯ 2 ◯ 3 ◯ 4 ◯ 5 ◯ 6 ◯ 7 ◯ 8 ◯ 9 ◯ 10 ◯ 11 ◯ 12
◯ 13 ◯ 14 ◯ 15 ◯ 16 ◯ 17 ◯ 18 ◯ 19 ◯ 20 ◯ 21 ◯ 22 ◯ 23 ◯ 24
◯ 25 ◯ 26 ◯ 27 ◯ 28 ◯ 29 ◯ 30 ◯ >30

i. Arterial Line Insertion

〇 0 〇 1 〇 2 〇 3 〇 4 〇 5 〇 6 〇 7 〇 8 〇 9 〇 10 〇 11 〇 12
〇 13 〇 14 〇 15 〇 16 〇 17 〇 18 〇 19 〇 20 〇 21 〇 22 〇 23 〇 24
〇 25 〇 26 〇 27 〇 28 〇 29 〇 30 〇 >30

Please rank how comfortable you are with the following procedures:

a. Abdominal Aortic Aneurism (AAA)

not comfortable at all — somewhat comfortable — very comfortable

(Place a mark on the scale above)

b. Deep Vein Thrombosis (DVT)

not comfortable at all — somewhat comfortable — very comfortable

(Place a mark on the scale above)

c. Arterial Flow

not comfortable at all — somewhat comfortable — very comfortable

(Place a mark on the scale above)

d. Peripheral IV access Insertion

not comfortable at all — somewhat comfortable — very comfortable

(Place a mark on the scale above)

e. Internal Jugular Central Line Insertion

not comfortable at all — somewhat comfortable — very comfortable

(Place a mark on the scale above)

f. Subclavian Central Line Insertion

not comfortable at all — somewhat comfortable — very comfortable

(Place a mark on the scale above)

g. Femoral Central Line Insertion

not comfortable at all — somewhat comfortable — very comfortable

(Place a mark on the scale above)

h. Peripherally Inserted Central Catheter (PICC) Insertion*

not comfortable at all — somewhat comfortable — very comfortable

(Place a mark on the scale above)

i. Arterial Line Insertion

not comfortable at all — somewhat comfortable — very comfortable

(Place a mark on the scale above)

POCUS Ocular Applications

Have you used POCUS for OCULAR applications when caring for patients in the past 6 months?

○ Yes ○ No

On average, how many times per week do you use POCUS for each of the applications listed below:

a. Optic Nerve Sheath Diameter

○ 0 ○ 1 ○ 2 ○ 3 ○ 4 ○ 5 ○ 6 ○ 7 ○ 8 ○ 9 ○ 10 ○ 11 ○ 12
○ 13 ○ 14 ○ 15 ○ 16 ○ 17 ○ 18 ○ 19 ○ 20 ○ 21 ○ 22 ○ 23 ○ 24
○ 25 ○ 26 ○ 27 ○ 28 ○ 29 ○ 30 ○ >30

b. Posterior Chamber

○ 0 ○ 1 ○ 2 ○ 3 ○ 4 ○ 5 ○ 6 ○ 7 ○ 8 ○ 9 ○ 10 ○ 11 ○ 12
○ 13 ○ 14 ○ 15 ○ 16 ○ 17 ○ 18 ○ 19 ○ 20 ○ 21 ○ 22 ○ 23 ○ 24
○ 25 ○ 26 ○ 27 ○ 28 ○ 29 ○ 30 ○ >30

Please rank how comfortable you are with the following procedures:

a. Optic Nerve Sheath Diameter

not comfortable at all | somewhat comfortable | very comfortable

(Place a mark on the scale above)

b. Posterior Chamber

not comfortable at all | somewhat comfortable | very comfortable

(Place a mark on the scale above)

POCUS Musculoskeletal/Soft Tissue Applications

Have you used POCUS for MUSCULOSKELETAL / SOFT TISSUE applications when caring for patients in the past 6 months?

◯ Yes ◯ No

On average, how many times per week do you use POCUS for each of the applications listed below:

a. Fractures

◯ 0 ◯ 1 ◯ 2 ◯ 3 ◯ 4 ◯ 5 ◯ 6 ◯ 7 ◯ 8 ◯ 9 ◯ 10 ◯ 11 ◯ 12
◯ 13 ◯ 14 ◯ 15 ◯ 16 ◯ 17 ◯ 18 ◯ 19 ◯ 20 ◯ 21 ◯ 22 ◯ 23 ◯ 24
◯ 25 ◯ 26 ◯ 27 ◯ 28 ◯ 29 ◯ 30 ◯ >30

b. Tendinopathies

◯ 0 ◯ 1 ◯ 2 ◯ 3 ◯ 4 ◯ 5 ◯ 6 ◯ 7 ◯ 8 ◯ 9 ◯ 10 ◯ 11 ◯ 12
◯ 13 ◯ 14 ◯ 15 ◯ 16 ◯ 17 ◯ 18 ◯ 19 ◯ 20 ◯ 21 ◯ 22 ◯ 23 ◯ 24
◯ 25 ◯ 26 ◯ 27 ◯ 28 ◯ 29 ◯ 30 ◯ >30

c. Cellulitis

◯ 0 ◯ 1 ◯ 2 ◯ 3 ◯ 4 ◯ 5 ◯ 6 ◯ 7 ◯ 8 ◯ 9 ◯ 10 ◯ 11 ◯ 12
◯ 13 ◯ 14 ◯ 15 ◯ 16 ◯ 17 ◯ 18 ◯ 19 ◯ 20 ◯ 21 ◯ 22 ◯ 23 ◯ 24
◯ 25 ◯ 26 ◯ 27 ◯ 28 ◯ 29 ◯ 30 ◯ >30

d. Foreign Body

◯ 0 ◯ 1 ◯ 2 ◯ 3 ◯ 4 ◯ 5 ◯ 6 ◯ 7 ◯ 8 ◯ 9 ◯ 10 ◯ 11 ◯ 12
◯ 13 ◯ 14 ◯ 15 ◯ 16 ◯ 17 ◯ 18 ◯ 19 ◯ 20 ◯ 21 ◯ 22 ◯ 23 ◯ 24
◯ 25 ◯ 26 ◯ 27 ◯ 28 ◯ 29 ◯ 30 ◯ >30

e. Abscess

◯ 0 ◯ 1 ◯ 2 ◯ 3 ◯ 4 ◯ 5 ◯ 6 ◯ 7 ◯ 8 ◯ 9 ◯ 10 ◯ 11 ◯ 12
◯ 13 ◯ 14 ◯ 15 ◯ 16 ◯ 17 ◯ 18 ◯ 19 ◯ 20 ◯ 21 ◯ 22 ◯ 23 ◯ 24
◯ 25 ◯ 26 ◯ 27 ◯ 28 ◯ 29 ◯ 30 ◯ >30

f. Joint Effusion

◯ 0 ◯ 1 ◯ 2 ◯ 3 ◯ 4 ◯ 5 ◯ 6 ◯ 7 ◯ 8 ◯ 9 ◯ 10 ◯ 11 ◯ 12
◯ 13 ◯ 14 ◯ 15 ◯ 16 ◯ 17 ◯ 18 ◯ 19 ◯ 20 ◯ 21 ◯ 22 ◯ 23 ◯ 24
◯ 25 ◯ 26 ◯ 27 ◯ 28 ◯ 29 ◯ 30 ◯ >30

g. Joint Injection

◯ 0 ◯ 1 ◯ 2 ◯ 3 ◯ 4 ◯ 5 ◯ 6 ◯ 7 ◯ 8 ◯ 9 ◯ 10 ◯ 11 ◯ 12
◯ 13 ◯ 14 ◯ 15 ◯ 16 ◯ 17 ◯ 18 ◯ 19 ◯ 20 ◯ 21 ◯ 22 ◯ 23 ◯ 24
◯ 25 ◯ 26 ◯ 27 ◯ 28 ◯ 29 ◯ 30 ◯ >30

h. Arthrocentesis

◯ 0 ◯ 1 ◯ 2 ◯ 3 ◯ 4 ◯ 5 ◯ 6 ◯ 7 ◯ 8 ◯ 9 ◯ 10 ◯ 11 ◯ 12
◯ 13 ◯ 14 ◯ 15 ◯ 16 ◯ 17 ◯ 18 ◯ 19 ◯ 20 ◯ 21 ◯ 22 ◯ 23 ◯ 24
◯ 25 ◯ 26 ◯ 27 ◯ 28 ◯ 29 ◯ 30 ◯ >30

Please rank how comfortable you are with the following procedures:

a. Fractures

not comfortable at all	somewhat comfortable	very comfortable

(Place a mark on the scale above)

b. Tendinopathies

not comfortable at all	somewhat comfortable	very comfortable

(Place a mark on the scale above)

c. Cellulitis

not comfortable at all	somewhat comfortable	very comfortable

(Place a mark on the scale above)

d. Foreign Body

not comfortable at all	somewhat comfortable	very comfortable

(Place a mark on the scale above)

e. Abscess

not comfortable at all	somewhat comfortable	very comfortable

(Place a mark on the scale above)

f. Joint Effusion

not comfortable at all	somewhat comfortable	very comfortable

(Place a mark on the scale above)

g. Arthrocentesis

not comfortable at all	somewhat comfortable	very comfortable

(Place a mark on the scale above)

Procedure-related complications: THORACENTESIS

1) Do you recall having any procedure-related complications while performing Thoracentesis?

○ No ○ Yes

Please select the complications you had while performing Thoracentesis.
(Please select all that apply)

☐ Iatrogenic pneumothorax
☐ Injury to intercostal artery or vein
☐ Other

How many Iatrogenic pneumothoraces have you had in the past year while performing Thoracenteses?

○ 1 ○ 2 ○ 3 ○ 4 ○ 5 ○ 6 ○ 7 ○ 8 ○ 9 ○ 10 ○ 11 ○ 12 ○ 13
○ 14 ○ 15 ○ 16 ○ 17 ○ 18 ○ 19 ○ 20 ○ >20

How many injuries to the intercostal artery or vein have you had in the past year?

○ 1 ○ 2 ○ 3 ○ 4 ○ 5 ○ 6 ○ 7 ○ 8 ○ 9 ○ 10 ○ 11 ○ 12 ○ 13
○ 14 ○ 15 ○ 16 ○ 17 ○ 18 ○ 19 ○ 20 ○ >20

Please describe the type of complication and number.

____

Was ultrasound used for guidance when you experienced these procedure-related complications?

○ No ○ Yes

Comments:

Procedure-related complications: PARACENTESIS

1) Do you recall having any procedure-related complications while performing Paracentesis (percutaneous abdominal drainage)?

○ No ○ Yes

Please select the complications you had while performing Paracentesis (percutaneous abdominal drainage). (Please select all that apply)

☐ Bowel injury
☐ Hemoperitoneum
☐ Other

How many bowel complications have you had in the past year?

○ 1 ○ 2 ○ 3 ○ 4 ○ 5 ○ 6 ○ 7 ○ 8 ○ 9 ○ 10 ○ 11 ○ 12 ○ 13
○ 14 ○ 15 ○ 16 ○ 17 ○ 18 ○ 19 ○ 20 ○ >20

How many hemoperitoneum complications have you had in the past year?

○ 1 ○ 2 ○ 3 ○ 4 ○ 5 ○ 6 ○ 7 ○ 8 ○ 9 ○ 10 ○ 11 ○ 12 ○ 13
○ 14 ○ 15 ○ 16 ○ 17 ○ 18 ○ 19 ○ 20 ○ >20

Please describe the type of complication and number.

____

Was ultrasound used for guidance when you experienced these procedure-related complications?

○ No ○ Yes

Comments:

Procedure-related complications: CENTRAL VENOUS CATHETER PLACEMENT

1) Do you recall having any procedure-related complications while performing Central venous catheter placement?

○ No ○ Yes

Please select the complications you had while performing Central venous catheter placement.
(Please select all that apply)

☐ Iatrogenic pneumothorax
☐ Injury or puncture of the common carotid artery
☐ Injury or puncture of the common femoral artery
☐ Other injury to a vein or artery
☐ Other

How many Iatrogenic pneumothoraces have you had in the past year while performing central venous catheter placement?

○ 1 ○ 2 ○ 3 ○ 4 ○ 5 ○ 6 ○ 7 ○ 8 ○ 9 ○ 10 ○ 11 ○ 12 ○ 13
○ 14 ○ 15 ○ 16 ○ 17 ○ 18 ○ 19 ○ 20 ○ >20

How many injuries or punctures of the common carotid artery have you had in the past year?

○ 1 ○ 2 ○ 3 ○ 4 ○ 5 ○ 6 ○ 7 ○ 8 ○ 9 ○ 10 ○ 11 ○ 12 ○ 13
○ 14 ○ 15 ○ 16 ○ 17 ○ 18 ○ 19 ○ 20 ○ >20

How many injuries or punctures of the common femoral artery have you had in the past year?

○ 1 ○ 2 ○ 3 ○ 4 ○ 5 ○ 6 ○ 7 ○ 8 ○ 9 ○ 10 ○ 11 ○ 12 ○ 13
○ 14 ○ 15 ○ 16 ○ 17 ○ 18 ○ 19 ○ 20 ○ >20

How many other injuries or punctures to veins or arteries have you had in the past year?

○ 1 ○ 2 ○ 3 ○ 4 ○ 5 ○ 6 ○ 7 ○ 8 ○ 9 ○ 10 ○ 11 ○ 12 ○ 13
○ 14 ○ 15 ○ 16 ○ 17 ○ 18 ○ 19 ○ 20 ○ >20

Please describe the type of complication and number.

____

Was ultrasound used for guidance when you experienced these procedure-related complications?

○ No ○ Yes

Comments:

Procedure-related complications: LUMBAR PUNCTURE

1) Do you recall having any procedure-related complications while performing Lumbar Puncture?

○ No ○ Yes

Please select the complications you had while performing Lumbar Punture.
(Please select all that apply)

☐ Subarachnoid hemorrhage
☐ Subdural hemorrhage
☐ Other

How many subarachnoid hemorrhages have you had in the past year?

○ 1 ○ 2 ○ 3 ○ 4 ○ 5 ○ 6 ○ 7 ○ 8 ○ 9 ○ 10 ○ 11 ○ 12 ○ 13
○ 14 ○ 15 ○ 16 ○ 17 ○ 18 ○ 19 ○ 20 ○ >20

How many subdural hemorrhages have you had in the past year?

○ 1 ○ 2 ○ 3 ○ 4 ○ 5 ○ 6 ○ 7 ○ 8 ○ 9 ○ 10 ○ 11 ○ 12 ○ 13
○ 14 ○ 15 ○ 16 ○ 17 ○ 18 ○ 19 ○ 20 ○ >20

Please describe the type of complication and number.

____

Was ultrasound used for guidance when you experienced these procedure-related complications?

○ No ○ Yes

Comments:

Point-of-Care Ultrasound (POCUS) Barriers

1) Do you encounter any barriers to your personal use of POCUS at your institution?
(Please select all that apply)

◯ Yes
◯ No

Ultrasound Machines
(Please select all that apply.)

☐ I can't find an ultrasound machine when I need one
☐ Setting up the ultrasound machine before procedures is too cumbersome
☐ Other

Clinician Factors
(Please select all that apply.)

☐ Using diagnostic POCUS to evaluate patients takes me too long
☐ I don't know how to operate the ultrasound machine
☐ I don't see any benefit to using POCUS in my practice
☐ I am concerned about becoming dependent on POCUS
☐ Time spent to use POCUS is not reimbursed or captured
☐ I do not have privileges to use POCUS
☐ I fear persecution for inappropriate use of POCUS
☐ I fear medical malpractice for missing findings on POCUS exams
☐ Other

Training
(Please select all that apply.)

☐ It's difficult to use POCUS skills learned in workshops in my clinical practice
☐ I don't know how to get trained in ultrasound use
☐ Training workshops are too expensive
☐ Availability of training workshops is limited
☐ Travel to training workshops is too expensive
☐ I don't have time to attend a POCUS training workshop
☐ Maintenance of POCUS skills is difficult
☐ Other

Facility Factors
(Please select all that apply.)

☐ My department does not have a clinical champion of POCUS
☐ My hospital does not have a way to archive ultrasound images
☐ My institution does not have POCUS policies
☐ My institution does not have a standard reporting form for POCUS
☐ Other

Please describe any other barriers to POCUS use.

2) Select the top three POCUS barriers in order of importance to you from the dropdown list:

Select the Greatest Barrier:

◯ I can't find an ultrasound machine when I need one
◯ Setting up the ultrasound machine before procedures is too cumbersome
◯ Using diagnostic POCUS to evaluate patients takes me too long
◯ I don't know how to operate the ultrasound machine
◯ I don't see any benefit to using POCUS in my practice
◯ I am concerned about becoming dependent on POCUS
◯ Time spent to use POCUS is not reimbursed or captured
◯ I do not have privileges to use POCUS
◯ I fear persecution for inappropriate use of POCUS
◯ I fear medical malpractice for missing findings on POCUS exams
◯ It's difficult to use POCUS skills learned in workshops in my clinical practice
◯ I don't know how to get trained in ultrasound use
◯ Training workshops are too expensive
◯ Availability of training workshops is limited
◯ Travel to training workshops is too expensive
◯ I don't have time to attend a POCUS training workshop
◯ Maintenance of POCUS skills is difficult
◯ My department does not have a clinical champion of POCUS
◯ My hospital does not have a way to archive ultrasound images
◯ My institution does not have POCUS policies
◯ My institution does not have a standard reporting form for POCUS
◯ Other

Select the Second Greatest Barrier:

◯ I can't find an ultrasound machine when I need one
◯ Setting up the ultrasound machine before procedures is too cumbersome
◯ Using diagnostic POCUS to evaluate patients takes me too long
◯ I don't know how to operate the ultrasound machine
◯ I don't see any benefit to using POCUS in my practice
◯ I am concerned about becoming dependent on POCUS
◯ Time spent to use POCUS is not reimbursed or captured
◯ I do not have privileges to use POCUS
◯ I fear persecution for inappropriate use of POCUS
◯ I fear medical malpractice for missing findings on POCUS exams
◯ It's difficult to use POCUS skills learned in workshops in my clinical practice
◯ I don't know how to get trained in ultrasound use
◯ Training workshops are too expensive
◯ Availability of training workshops is limited
◯ Travel to training workshops is too expensive
◯ I don't have time to attend a POCUS training workshop
◯ Maintenance of POCUS skills is difficult
◯ My department does not have a clinical champion of POCUS
◯ My hospital does not have a way to archive ultrasound images
◯ My institution does not have POCUS policies
◯ My institution does not have a standard reporting form for POCUS
◯ Other

Select the Third Greatest Barrier:

◯ I can't find an ultrasound machine when I need one
◯ Setting up the ultrasound machine before procedures is too cumbersome
◯ Using diagnostic POCUS to evaluate patients takes me too long
◯ I don't know how to operate the ultrasound machine
◯ I don't see any benefit to using POCUS in my practice
◯ I am concerned about becoming dependent on POCUS
◯ Time spent to use POCUS is not reimbursed or captured
◯ I do not have privileges to use POCUS
◯ I fear persecution for inappropriate use of POCUS
◯ I fear medical malpractice for missing findings on POCUS exams
◯ It's difficult to use POCUS skills learned in workshops in my clinical practice
◯ I don't know how to get trained in ultrasound use
◯ Training workshops are too expensive
◯ Availability of training workshops is limited
◯ Travel to training workshops is too expensive
◯ I don't have time to attend a POCUS training workshop
◯ Maintenance of POCUS skills is difficult
◯ My department does not have a clinical champion of POCUS
◯ My hospital does not have a way to archive ultrasound images
◯ My institution does not have POCUS policies
◯ My institution does not have a standard reporting form for POCUS
◯ Other

3) Based on your own personal experiences with using POCUS, do you feel POCUS improves the patient experience?

◯ Yes
◯ No

Please select improvements to patient experience you feel POCUS provides:
(Please select all that apply)

☐ Improves patient's understanding of his/her condition
☐ Improves patient's confidence in his/her providers
☐ Improves communication between patients and providers
☐ Improves doctor-patient relationships because providers spend more time at the bedside
☐ Improves relationships with patients' families
☐ Reduces patient discomfort or pain
☐ Reduces unnecessary consultations for the patient
☐ Reduces unnecessary imaging or testing of the patient
☐ Other

Please list other improvements.

4) Have you had any POCUS training in addition to the SimLEARN training course in Orlando?

◯ Yes
◯ No

Please select previous POCUS training.
(Please select all that apply)

- ☐ Medical School
- ☐ Residency/Fellowship
- ☐ Workshop by professional society
- ☐ Workshop by your institution
- ☐ Workshop by private education company or industry sponsored
- ☐ Bedside training from a colleague
- ☐ Computer-based training (online, DVD, or app) or printed materials
- ☐ Other

Please list.

____

System Factors:

Complete the following questions to the best of your knowledge about your facility.

1) What percentage of providers in your specialty use POCUS currently at your VA facility?

◯ 0 - 25%
◯ 26 - 50%
◯ 51 - 75%
◯ 76 - 100%

2) What other specialties at your facility use POCUS?
(Please select all that apply.)

☐ Emergency Medicine
☐ Internal Medicine
☐ Critical Care
☐ Pulmonary
☐ Rheumatology
☐ Endocrinology
☐ Nephrology
☐ Gastroenterology
☐ Anesthesiology
☐ Orthopedic Surgery
☐ Vascular Surgery
☐ General Surgery
☐ Trauma Surgery
☐ Urology
☐ Ophthalmology
☐ Other
☐ I do not know who uses POCUS at my facility.

Please specify:

____

3) Does your facility provide POCUS training for its providers?

◯ Yes ◯ No

What type of POCUS training currently exists at your facility?
(Please select all that apply.)

☐ VA-sponsored onsite CME course
☐ Non-VA sponsored CME course (e.g., academic affiliate, self-sponsored, corporate sponsor)
☐ Other

Please describe:

____

4) Do any of these core VHA National POCUS Training Program faculty members work in your hospital?
(Please select all that apply.)

☐ Nilam Soni
☐ Jeremy Boyd
☐ Christopher Scott
☐ Rahul Khosla
☐ Nima Afsar
☐ Megan Core
☐ Other

Please list.

____

5) What brands of ultrasound machine(s) do you use for POCUS at your facility?
(Please select all that apply.)

☐ I do not know what brands of ultrasound machines are at my institution.
☐ Esaote
☐ GE
☐ Mindray
☐ Philips
☐ Samsung
☐ Siemens
☐ Sonosite
☐ Terason
☐ Toshiba
☐ Zonare
☐ Other

Please list:

____

How many ultrasound machines are available in these departments?

Emergency Department:

◯ I don't know
◯ 0
◯ 1
◯ 2
◯ 3
◯ 4
◯ 5
◯ 6
◯ 7
◯ 8
◯ 9
◯ 10
◯ 11
◯ 12
◯ 13
◯ 14
◯ 15
◯ 16
◯ 17
◯ 18
◯ 19
◯ 20
◯ >20

Medical Intensive Care Unit (ICU)

◯ I don't know
◯ 0
◯ 1
◯ 2
◯ 3
◯ 4
◯ 5
◯ 6
◯ 7
◯ 8
◯ 9
◯ 10
◯ 11
◯ 12
◯ 13
◯ 14
◯ 15
◯ 16
◯ 17
◯ 18
◯ 19
◯ 20
◯ >20

Surgical Intensive Care Unit(s) (ICU)

◯ I don't know
◯ 0
◯ 1
◯ 2
◯ 3
◯ 4
◯ 5
◯ 6
◯ 7
◯ 8
◯ 9
◯ 10
◯ 11
◯ 12
◯ 13
◯ 14
◯ 15
◯ 16
◯ 17
◯ 18
◯ 19
◯ 20
◯ >20

Inpatient Medical Ward

◯ I don't know
◯ 0
◯ 1
◯ 2
◯ 3
◯ 4
◯ 5
◯ 6
◯ 7
◯ 8
◯ 9
◯ 10
◯ 11
◯ 12
◯ 13
◯ 14
◯ 15
◯ 16
◯ 17
◯ 18
◯ 19
◯ 20
◯ >20

Surgical Medical Ward

◯ I don't know
◯ 0
◯ 1
◯ 2
◯ 3
◯ 4
◯ 5
◯ 6
◯ 7
◯ 8
◯ 9
◯ 10
◯ 11
◯ 12
◯ 13
◯ 14
◯ 15
◯ 16
◯ 17
◯ 18
◯ 19
◯ 20
◯ >20

Ambulatory Procedure Clinic

- ◯ I don't know
- ◯ 0
- ◯ 1
- ◯ 2
- ◯ 3
- ◯ 4
- ◯ 5
- ◯ 6
- ◯ 7
- ◯ 8
- ◯ 9
- ◯ 10
- ◯ 11
- ◯ 12
- ◯ 13
- ◯ 14
- ◯ 15
- ◯ 16
- ◯ 17
- ◯ 18
- ◯ 19
- ◯ 20
- ◯ >20

Operating Room Machine(s)

- ◯ I don't know
- ◯ 0
- ◯ 1
- ◯ 2
- ◯ 3
- ◯ 4
- ◯ 5
- ◯ 6
- ◯ 7
- ◯ 8
- ◯ 9
- ◯ 10
- ◯ 11
- ◯ 12
- ◯ 13
- ◯ 14
- ◯ 15
- ◯ 16
- ◯ 17
- ◯ 18
- ◯ 19
- ◯ 20
- ◯ >20

Ambulatory Procedure Clinic

◯ I don't know
◯ 0
◯ 1
◯ 2
◯ 3
◯ 4
◯ 5
◯ 6
◯ 7
◯ 8
◯ 9
◯ 10
◯ 11
◯ 12
◯ 13
◯ 14
◯ 15
◯ 16
◯ 17
◯ 18
◯ 19
◯ 20
◯ >20

SYSTEM FACTORS

6) POCUS Locations and Ultrasound Machine Access

	Dedicated Machine	Shared Machine	I'm not sure
Emergency Department	◯	◯	◯
Medical Intensive Care Units (ICU)	◯	◯	◯
Surgical Intensive Care Unit(s) (ICU)	◯	◯	◯
Inpatient Medical Ward	◯	◯	◯
Inpatient Surgical Ward	◯	◯	◯
Ambulatory Procedure Clinic	◯	◯	◯
Operating Room	◯	◯	◯

Other comments regarding routine POCUS use and access between your facility's departments:

7) Does your facility require INITIAL demonstration of individual provider competency in POCUS to grant privileges?

◯ Yes
◯ No
◯ I don't know

Select the demonstration methods your facility uses to initially determine individual provider POCUS competency. (Select all methods that apply.)

☐ Focused Professional Practice Evaluation (FPPE) (Prospective, Concurrent, Retrospective)
☐ Attestation/verification of basic competency obtained during graduate medical education training program (residency/fellowship)
☐ Personal attestation of basic competency
☐ Completion of an accredited POCUS continuing medical education (CME) course with hands-on sessions
☐ Demonstrated/tracked at academic affliiate
☐ Documented minimum number of cases (e.g., 25 cases per application)
☐ Simulation Demonstration
☐ Live Proctoring
☐ Other

Please describe other methods or comments regarding initial privileging.

8) Does your facility require ONGOING demonstration of individual provider competency in POCUS to grant privileges?

◯ Yes
◯ No
◯ I don't know

Select the demonstration methods your facility uses to determine individual provider ongoing POCUS competency. (Select all methods that apply.)

☐ Ongoing Professional Practice Evaluation (OPPE) (Prospective, Concurrent, Retrospective)
☐ Attestation/verification of competency from supervisor/service chief
☐ Personal attestation of competency
☐ Completed/tracked at academic affiliate
☐ Documented minimum number of cases per reappointment cycle (e.g. 5 cases per application)
☐ Simulation Demonstration
☐ Live Proctoring
☐ Other

Please describe other methods or comments regarding ongoing privileges.

9) Does your facility do periodic quality assurance reviews of POCUS cases?

◯ Yes
◯ No
◯ I don't know

Select the type of quality assurance review(s) your facility uses for POCUS cases.
(Please select all that apply.)

☐ Retrospective review of cases
☐ Simulation Demonstration
☐ Live Proctoring
☐ Other

Please list:

10) Are POCUS images recorded and saved?

◯ Yes
◯ No
◯ I don't know

Where are they saved?

☐ On the machine
☐ Electronic medical record
☐ VA server (other than electronic medical record)
☐ Non-VA server
☐ Other

Please specify:

Are the recorded images reviewed by a second level POCUS designee/expert?

◯ I don't know
◯ Yes
◯ No

Is there a protocol or agreement for preliminary vs. official interpretation?

◯ I don't know
◯ Yes
◯ No

11) How is workload captured for POCUS?
(Please select all that apply.)

☐ Encounters are generated and providers select CPT codes
☐ Coders review documentation and select appropriate codes for encounters
☐ Workload is NOT captured for POCUS
☐ Other

Please describe.

Please provide any additional comments or clarifications about your survey responses:
