# Supplementary material for: Paracentesis complication rates and use of ultrasound: impact of a point-of-care ultrasound training course in the veterans affairs healthcare system
Source: BMC Med Educ. 2025 Aug 12;25:1161. doi: 10.1186/s12909-025-07656-z (PMC12341121; doi:10.1186/s12909-025-07656-z)
Supplement: Supplementary file 3 — Supplementary Material 3: Additional File 3. Paracentesis Complications and Total Number of Paracenteses during Pre-Intervention, Intervention, and Post-Intervention Periods [file 12909_2025_7656_MOESM3_ESM.docx]

**Additional File 3. Paracentesis Complications and Total Number of Paracenteses during Pre-Intervention, Intervention, and Post-Intervention Periods**

| **Facility Group** | **Pre-Intervention**  (October 2015 to September 2016) | | **Intervention**  (October 2016 to September 2019) | | | | | |
| --- | --- | --- | --- | --- | --- | --- | --- | --- |
|  | **FY16** | | **FY17** | | **FY18** | | **FY19** | |
|  | Comp. | Total | Comp. | Total | Comp. | Total | Comp. | Total |
| Trained | 5 | 1403 | 0 | 1160 | 4 | 1101 | 6 | 1287 |
| Matched | 11 | 2886 | 4 | 2712 | 5 | 2751 | 4 | 2219 |

Comp, complications; FY, fiscal year.

| **Facility Group** | **Post-Intervention**  (October 2019 to March 2025) | | | | | | | | | | | |
| --- | --- | --- | --- | --- | --- | --- | --- | --- | --- | --- | --- | --- |
|  | **FY20** | | **FY21** | | **FY22** | | **FY23** | | **FY24** | | **FY25 (Q1-2)** | |
|  | Comp. | Total | Comp. | Total. | Comp. | Total. | Comp. | Total | Comp. | Total | Comp. | Total |
| Trained | 6 | 1488 | 2 | 1348 | 4 | 1517 | 5 | 1393 | 4 | 1555 | 1 | 712 |
| Matched | 3 | 1929 | 4 | 2436 | 3 | 2388 | 4 | 2006 | 5 | 1913 | 1 | 863 |

Comp, complications; FY, fiscal year.
