# Supplementary material for: Paracentesis complication rates and use of ultrasound: impact of a point-of-care ultrasound training course in the veterans affairs healthcare system
Source: BMC Med Educ. 2025 Aug 12;25:1161. doi: 10.1186/s12909-025-07656-z (PMC12341121; doi:10.1186/s12909-025-07656-z)
Supplement: Supplementary file 4 — Supplementary Material 4: Additional File 4. Use of Ultrasound Guidance by Radiology for Paracentesis from October 2015 to March 2025 [file 12909_2025_7656_MOESM4_ESM.docx]

**Additional File 4. Use of Ultrasound Guidance by Radiology for Paracentesis from October 2015 to March 2025**

POCUS, point-of-care ultrasound
