# Supplementary material for: Paracentesis complication rates and use of ultrasound: impact of a point-of-care ultrasound training course in the veterans affairs healthcare system
Source: BMC Med Educ. 2025 Aug 12;25:1161. doi: 10.1186/s12909-025-07656-z (PMC12341121; doi:10.1186/s12909-025-07656-z)
Supplement: Supplementary file 5 — Supplementary Material 5: Additional File 5. Paracentesis Complications per Coding Data [file 12909_2025_7656_MOESM5_ESM.docx]

**Additional File 5. Paracentesis Complications per Coding Data**

| **Procedure** | **Complication Type** | **Trained Facilities** | **Matched Facilities** | **p-value** |
| --- | --- | --- | --- | --- |
| Paracentesis | Bleeding  Bowel Injury | 37 (100%)  0 (0%) | 44 (100%)  0 (0%) | NS |

NS, not significant.
